# Autoimmune hypophysitis and viral infection in a pregnant woman: a challengeable case

**DOI:** 10.11604/pamj.2020.36.28.22454

**Published:** 2020-05-21

**Authors:** Kais Benabderrahim

**Affiliations:** 1 Department of Ophthalmology, University Hospital of Medenine, Faculty of Medicine, Sfax University, Tunisia

**Keywords:** Autoimmune hypophysitis, viral infection, pregnancy

## Abstract

The aim of this study is to report a challengeable and rare case of autoimmune hypophysitis possibly induced by viral infections. A young pregnant female developed optic neuropathy due to enlarged sellar mass responsible for hypopituitarism. Investigations exclude neoplasia and systemic diseases. High level of sedimentation rate and magnetic resonance imaging (MRI) findings supported the diagnosis of autoimmune hypophysitis. The patient reported a history of bronchitis treated with antibiotics and corticosteroids and positive serologies for hepatitis B antigen (Hbs antigen), herpes simplex 1 and rubella. Final examination showed complete recovery of visual function and sellar archnoidocele after antiviral treatment and mild dose of corticosteroids.

## Introduction

Autoimmune hypophysitis (AH) is a rare autoimmune inflammatory disorder of the pituitary gland appeared as an enlarged mass resembling macroadenoma [1,2]. Treatment of this entity was corticosteroid [1,2]. Viral infections were widespread in all the world. They may induce autoimmune diseases in such conditions [3]. Preview experimental studies and reviews reported factors inducing autoimmune hypophysitis including rubella and other viral infections [3-5]. We describe an unusual case of AH presumed to be induced by viral infections.

## Patient and observation

**Figure 1:**
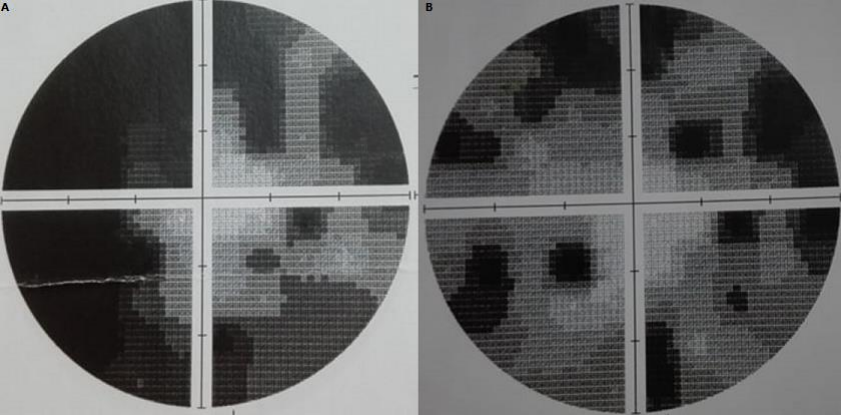
Wide defect in visual field of the right eye (A) that rapidly improved after one day of treatment (B)

**Figure 2:**
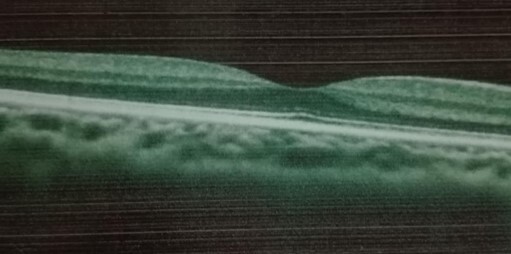
Normal macular region in the right eye showed with optical coherence tomography

**Figure 3:**
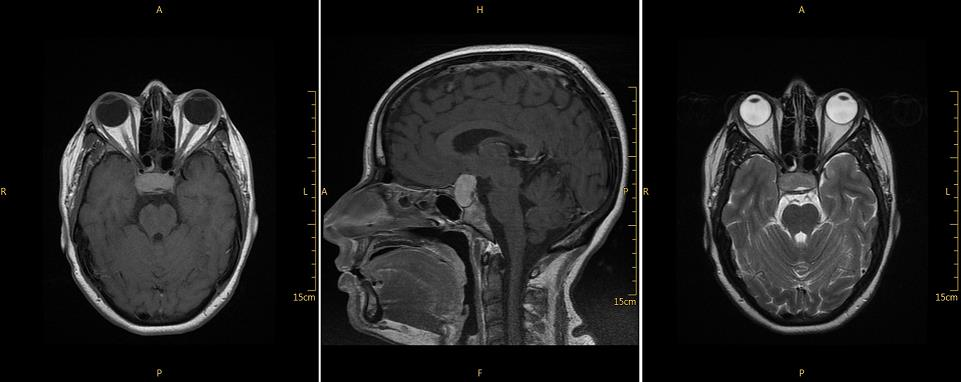
MRI revealed large sellar mass with lateral and supra sellar extension compressed optic chiasma and intense homogenous enhancement

A 25-year-old female, in the third trimester (38 weeks) of pregnancy, presented with sudden blurred vison in her right eye, headache and vomiting. She had a 1-month past history of oral herpetic infection improved by local acyclovir. She reported a history of methylprednisolone and antibiotics treatment for influenzae and bronchitis 20 days ago. Pregnancy follow-up revealed positive Hbs antigen and positive IgG level for rubella as a sign of old immunity. Ophthalmological examination found myopic eyes, normal ocular pressure, visual acuity of 1/10 in the right eye and 10/10 in the left eye and relative pupillary defect implied right optic neuropathy. Fundus showed myopic findings without any optic disc swelling. Visual field exam revealed large defect in the right eye (Figure 1). Optical coherence tomography showed no macular pathology (Figure 2). B-scan echography showed normal findings. General examination and blood investigations excluded toxemia and preeclampsia (blood pressure=10/6, platelet count =273000/mm3, alanine transaminase =17 UI/l, aspartate transaminase =28 UI/l, azotemia = 0.11 g/l, creatinine =4.1 mg/l). Biological investigations revealed a high level of erythrocyte sedimentation rate (ESR) (109 mm/hour), leukocytes=5400/mm3 (neutrphils=65%, lymphocytes=24%, monocytes=9%), glycemia = 0.76 g/l, proteinuria = 0.09 g/L, a normal level of C reactive protein (CRP) (2mg/dL) and normal level of gamma globulin. Magnetic resonance imaging (MRI) showed a large pituitary mass (diameter = 27 mm) resembling macroadenoma with suprasellar and lateral extension responsible for moderate compression of optic chiasma with an intense homogenous post-contrast enhancement and stalk thickening (Figure 3). Hormonal profile revealed hypopituitarism included low levels of free thyroxine (4.26 pmol/L), TSH (0.21 μUI/mL) and diabetes insipidus (polyuro-polydipsic syndrome). Cortisol (79 ng/mL) and prolactin (5 ng/mL) were mildly affected. The diagnosis of compressive optic neuropathy results from autoimmune hypophysitis was done. Etiologic investigations revealed high level of antinuclear antibodies (1/100) in the blood without any other features of systemic disease or neoplasic pathology. Serologic test showed positive level of IgG for Herpes simplex 1. Parenteral acyclovir was started at a dose of 200 mg twice a day. A single 4 mg-dose of methylprednisolone was administrated just before cesarean performed after 7 days. Rapid improvement of visual acuity (4/10) and regression of field defect were noted in the first day (Figure 1). Complete recovery of visual acuity was rapid. Follow-up MRI showed arachnoidocele of sella, undisplaced shalk, decreased size of antehypophysis, normal enhancement of the ante and post hypophysis. At the final examination, recovery of hormonal profile was noted without any recurrence in 3 years.

## Discussion

In this report, clinical features, biological findings, and MRI supported the diagnosis of optic neuropathy due to an AH induced by viral infections. AH is a rare condition, usually seen in pregnancy or post-partum, characterized by sellar mass resembling adenoma and responsible for headache, visual impairment and variable degree of hypopituitarism [1,6]. Biologic features of AH included a high level of sedimentation rate [2,7]. In contrast to macroadenoma, sellar mass is symmetrical and homogeneous with thickened but undisplaced stalk [2,6]. Actually, studies strongly suggested a relationship between neurologic involvement of autoimmunity and viral infections including herpes simplex virus, measles, influenza virus, rubella, hepatitis C virus, and Epstein-barr Virus [3]. At least 4 viruses were encountered in this patient. The occurrence of clinical manifestations in a patient under corticosteroids supports the diagnosis of virus infection or viral induction of the disease. In addition, previous Cochrane review concluded that adjunctive corticosteroid therapy in the treatment of influenza may be associated with increased mortality[8]. We give more attention to herpetic infection that was prevalent and may leading to severe cases of morbidity and neonatal mortality [9]. Authors reported that although there is no demonstrated benefit for prophylactic treatment in reducing the risk of neonatal herpes, anti-viral prophylaxis is recommended after 36 weeks of amenorrhea to limit the need for caesarean section [10]. The recommended antivirals are acyclovir at a dosage of 400mg until delivery. All data supported our use of acyclovir not only to improve the visual outcome but also to reduce neonatal complications [9,10]. Estimated incidence of AH is one in nine million per year. It occurs predominantly in young pregnant females, especially in the peripartum period [1,2]. It is classified as a primary hypophysitis that remained as a diagnosis of exclusion. Diagnosis of AH was considered when neoplasic pathology and secondary causes were ruled out [1,2]. Biopsy was made to distinguish between neoplastic and inflammatory causes. It requires surgery that is not always possible or necessary for an effective clinical management of the pathology [2,6].

Actually, hypophysitis can be differentiated from macroadenoma on MRI. Hypophysitis is a symmetrical, intensely and homogenously enhancing mass, with thickened but undisplaced stalk and intact sellar floor in contrast to typical findings with pituitary tumors [1,2,6]. Primary hypophysitis was classified into Lymphocytic Hypophysitis (AH) seen in the pregnant woman, granulomatous hypohysitsis due to granulomatous disease as sarcoidosis and tuberculosis, Xanthomatous Hypophysitis, IgG4-related disease and immune checkpoint therapy related hypophysitis induced by immunomodulatory therapy for cancer [2]. In this case, MRI findings in additions to clinical presentation of the disease, laboratory investigations and score described by Gutenberg et al supported the diagnosis of Autoimmune hypophysitis [1,2,6]. The clinical features included headache, variable degrees of hypopituitarism (cortisol and thyroid axes were commonly affected) and frequently diabetes insipidus. Visual disorder was common (33%) but optic neuropathy was rarely seen [1,2,6]. Management of patients with AH consisted on replacement of hormonal deficiency and corticosteroids [2]. Authors reported the efficacy of pulses of corticosteroids (120 mg a day) in the management of AH. However, recurrence rate (38%) has been reported to be high and it highlights the limitations of this treatment [1]. Viral infection may explain the mechanism inducing this idiopathic disease. A previous experimental report concluded that pituitary gland may be susceptible to T cell mediated pathology after immunization of a virus expressing soluble pituitary gland antigen [4]. Besides, authors reported that rubella virus proteins extracted from vaccina virus recombinants induced AH [5]. In our case, at least three viral infections were encountered in the patient. Blurred vision seen after corticosteroids and rapid improvement with antivirals support this mechanism.

## Conclusion

Autoimmune hypophysitis should be considered in any pregnant woman with sellar mass. Viral induction of this disease should be considered before starting corticosteroids.

## Competing interests

The author declares no competing interests.
